# Community Water Trihalomethanes and Chronic Kidney Disease

**DOI:** 10.1001/jamanetworkopen.2025.18513

**Published:** 2025-07-02

**Authors:** Danielle N. Medgyesi, Sumit Mohan, Komal Bangia, Emma S. Spielfogel, Maya Spaur, Jared A. Fisher, Jessica M. Madrigal, Ana Navas-Acien, Laura E. Beane Freeman, Rena R. Jones, Mary H. Ward, James V. Lacey, Tiffany R. Sanchez

**Affiliations:** 1 Department of Environmental Health Sciences, Mailman School of Public Health, Columbia University, New York, New York; 2 Division of Nephrology, Department of Medicine, Columbia University Medical Center, New York, New York; 3 Department of Epidemiology, Mailman School of Public Health, Columbia University, New York, New York; 4 Community and Environmental Epidemiology Research Branch, Office of Environmental Health Hazard Assessment, Oakland, California; 5 Division of Health Analytics, Department of Computational and Quantitative Medicine, Beckman Research Institute, City of Hope, Duarte, California; 6 Occupational and Environmental Epidemiology Branch, Division of Cancer Epidemiology and Genetics, National Cancer Institute, Rockville, Maryland; 7 Department of Epidemiology, Colorado School of Public Health, University of Colorado Anschutz Medical Campus, Aurora

## Abstract

**Importance:**

Over 90% of the US population relies on community water supplies (CWS), which generally use chlorine for disinfection. Trihalomethanes are regulated disinfection byproducts associated with bladder cancer and adverse birth outcomes. Animal studies report trihalomethanes, especially brominated compounds, may damage kidney function, but epidemiologic research is limited.

**Objective:**

To evaluate long-term exposure to trihalomethanes in residential CWS and its association with chronic kidney disease (CKD) risk.

**Design, Setting, and Participants:**

The California Teachers Study (CTS) is an ongoing prospective cohort of female teachers and administrators enrolled between 1995 and 1996 with data linked to mortality and health care records. This cohort study analyzed CTS data from January 1, 2005, once CKD diagnostic coding was adopted, through December 31, 2018. Statistical analysis was conducted from July 2023 to December 2024.

**Exposures:**

Residence time-weighted mean concentrations of 4 trihalomethanes, including 3 brominated trihalomethanes and chloroform, were calculated using annual measurements from CWS serving participants’ homes from 1995 to 2005. Uranium and arsenic (potentially nephrotoxic metals, previously evaluated in the cohort) from CWS were included as part of a g-computation mixture analysis.

**Main Outcomes and Measures:**

Cases of moderate (stage 3) to end-stage CKD were identified with diagnostic codes or dialysis-related procedures. Mixed-effects multivariable-adjusted Cox proportional hazards regression models were used to estimate hazard ratios (HRs) and 95% CIs for CKD by exposure to trihalomethane levels (US maximum contaminant level of 80 μg/L).

**Results:**

The study sample included 89 320 female participants (median age, 50 years [IQR, 43-61 years]) with 6242 CKD cases. Median concentrations were 5.5 μg/L (IQR, 0.5-24.1 μg/L; 95th percentile, 57.8 μg/L) for total trihalomethanes and 2.7 μg/L (IQR, 0.7-11.3 μg/L; 95th percentile, 30.0 μg/L) for brominated trihalomethanes. In flexible spline-based models, a clear exposure-response association was observed between trihalomethanes and CKD risk, with the highest risk for brominated trihalomethanes. The HRs for CKD risk associated with brominated trihalomethanes at the highest 2 exposure categories (75th percentile and at or above the 95th percentile) were 1.23 (95% CI, 1.13-1.33) and 1.43 (95% CI, 1.23-1.66), respectively (*P* < .001 for trend). Brominated trihalomethanes were the largest contributor (52.9%) to the association of the overall mixture with CKD risk, followed by uranium (35.4%), arsenic (6.2%), and chloroform (5.5%).

**Conclusions and Relevance:**

In this prospective cohort study of California female teachers, exposure to trihalomethane concentrations less than 80 μg/L (US current standard) increased CKD risk, particularly brominated trihalomethanes, which are not separately regulated in community water. The findings may have public health implications given the widespread use of water chlorination and growing burden of CKD.

## Key Points

**Question:**

Are trihalomethanes from community water supplies associated with chronic kidney disease (CKD) risk in adults?

**Findings:**

In this cohort study of 89 320 female teachers and school administrators in California, exposure to higher levels of trihalomethanes, especially brominated compounds, was associated with higher risk of CKD over the follow-up. The risk of CKD was greater for long-term exposure to mean brominated trihalomethane concentration of 30.0 μg/L or more (≥95th percentile) compared with less than 0.7 μg/L (<25th percentile).

**Meaning:**

The findings suggest trihalomethane exposure at levels below current regulatory standards, particularly brominated compounds, which are not separately regulated, may increase the risk of CKD.

## Teaser

This cohort study examines whether long-term exposure to trihalomethanes in community water supplies was associated with increased risk of chronic kidney disease in a cohort of female adults in California.

## Introduction

Chronic kidney disease (CKD)—defined by the gradual loss of kidney function—is a prevalent, underrecognized condition, ranked among the top 10 causes of death in the US.^1,2,3^ Worldwide, CKD prevalence is rising at a rapid rate, outpacing that of other major chronic diseases including diabetes and cardiovascular disease.^4^ Growing evidence suggests environmental exposures, including water contaminants, are modifiable contributors to the development of CKD.^5,6^

Community water supplies (CWS) serve more than 90% of the US population.^7^ The use of chlorine to remove harmful microbes is a long-standing public health practice adopted by most CWS.^8,9^ An unintended consequence of chlorination is the formation of disinfection byproducts, including trihalomethanes (THMs), which occur in the presence of organic matter.^10,11,12^ The additional presence of bromide, common in saltwater and coastal regions, creates brominated THMs.^10,13,14^ Threats to water quality from climate change, including rising temperatures, coastal flooding, acidification, and organic matter from pollution and wildfires, are likely to exacerbate THM formation.^14,15,16,17,18^

THMs have been associated with adverse health consequences and extensively studied in the context of bladder cancer and restricted fetal growth.^19,20,21,22^ The US Environmental Protection Agency (EPA) regulates the sum of 4 THMs—chloroform and 3 brominated compounds—in CWS at a maximum contaminant level (MCL) of 80 μg/L.^7^ Nonenforceable public health goals, based solely on scientific evidence, have been set for each of the 4 individual THM compounds, with an MCL goal of 0 μg/L for bromodichloromethane and bromoform, reflecting their greater toxicologic potential at lower doses.^7,23^

Animal studies have documented the nephrotoxic effects of THMs, including proximal convoluted tubular injury, impaired concentrating ability, and decreased glomerular filtration rate (GFR).^24,25,26,27,28,29,30,31^ Studies of kidney toxic effects found brominated compounds (particularly bromodichloromethane) are more nephrotoxic than chloroform.^29,30^ These studies, conducted decades ago, provide plausibility for THM nephrotoxic effects, yet there is a notable scarcity of epidemiologic studies.^32^ In the 2003-2012 National Health and Nutrition Examination Survey (NHANES), higher blood levels of bromodichloromethane and chloroform were associated with lower levels of estimated GFR.^32^

The current study, conducted using data from the California Teachers Study (CTS)—a large prospective cohort of females with incident CKD data collected for over 10 years—seeks to evaluate whether CKD risk associated with prolonged THM exposure is (1) observed below the current regulatory THM limit and (2) differential by the presence of brominated species. Using a mixture approach, we estimated the comparative contribution of brominated THMs vs chloroform to CKD risk, alongside 2 other potentially nephrotoxic water contaminants, uranium and arsenic, previously evaluated in the cohort.^33^

## Methods

### Study Population and Outcome Ascertainment

This cohort study used data from the CTS, which includes 133 477 females enrolled between 1995 and 1996 (aged 22 to 104 years at enrollment) who were recruited from the California State Teachers Retirement System and completed a mailed questionnaire.^34^ The cohort has been routinely followed up since inception and includes sociodemographic, health, and lifestyle information from 5 follow-up questionnaires. Self-reported race and ethnicity were collected in the enrollment survey and coded according to the National Institutes of Health reporting requirements (eTable 1 in Supplement 1); categories were Asian, Black or African American, Hispanic or Latina, White, and unknown or additional categories (due to limited numbers, we consolidated American Indian or Alaska Native, Native Hawaiian or Other Pacific Islander, or multiracial but recognize that each of these groups is unique and does not share a common identity). The CTS has been approved by the institutional review board at City of Hope, and participants provided written informed consent at enrollment. The current study was approved by the institutional review boards of City of Hope and Columbia University, with written informed consent obtained via completion of the mailed questionnaire. We followed the Strengthening the Reporting of Observational Studies in Epidemiology (STROBE) reporting guideline.

Participants in the CTS have been annually linked to inpatient hospitalization records and, since 2005, emergency department visits and ambulatory surgeries from the California Department of Health Care Access and Information. They have also been linked to mortality records from the State of California mortality files, the Social Security Administration Death Master File, and the National Death Index.

Follow-up for this analysis began on January 1, 2005, excluding prior years when CKD capture was incomplete and once diagnostic codes for CKD were adopted.^35^ Our primary outcome, referred to as moderate or greater CKD, included claims for CKD stages 3 to 5, end-stage kidney disease (ESKD), or dialysis-related procedures (eTable 2 in Supplement 1). We also evaluated secondary definitions: (1) advanced CKD cases (stages 4-5 and ESKD) and (2) all CKD cases (stages 1-5 and ESKD). Evaluation of severe CKD was limited by fewer cases and of all CKD by the underrecognition of earlier stages in claims data.^36^ Participant follow-up concluded at the earliest of the following dates: observed CKD event, death, relocation out of California (since this study relied on statewide data), or December 31, 2018, whichever came first. This study excluded participants who died or moved out of California before January 1, 2005, had a poorly geocoded enrollment address,^37^ lived outside CWS boundaries (assumed to be private well users), had missing THM data, had a prevalent CKD diagnosis (based on administrative records), were aged 85 years or older at enrollment, or were missing covariate information (eFigure 1 in Supplement 1).

### Water Trihalomethanes Exposure Assessment

**Figure 1.**
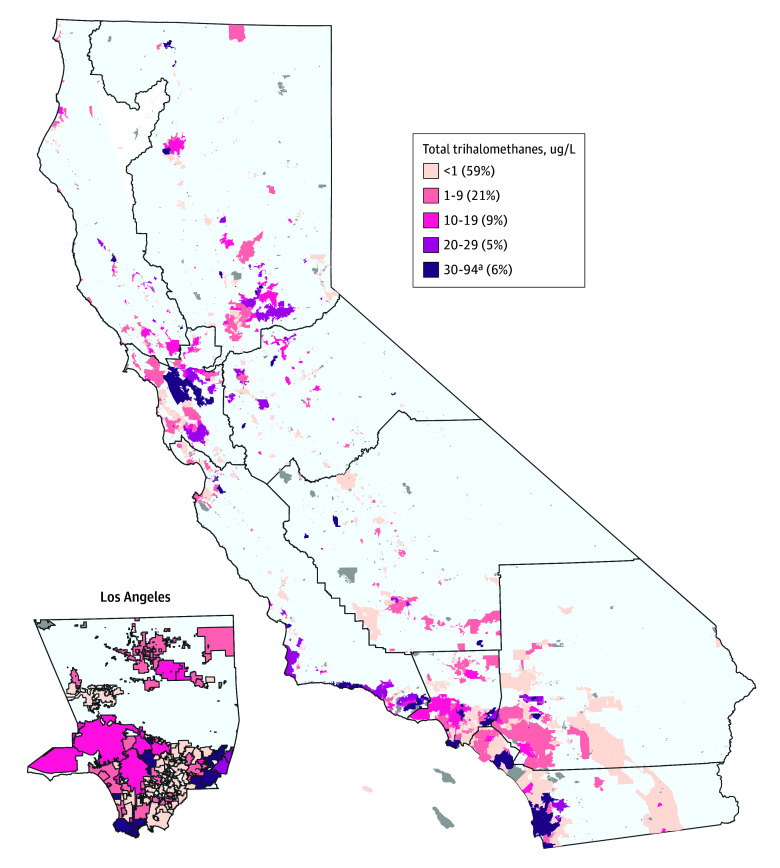
Map of California Community Water Supplies and Average Total Trihalomethane Concentrations for the Exposure Period (1995-2005) Includes 2933 community water supplies. Gray shading indicates supplies that had missing data or were not linked to California Teacher Study participants. The 10 census regions of California are outlined and labeled. ^a^Two community water supplies had concentrations of 80 μg/L or higher, the US Environmental Protection Agency maximum contaminant level.

We estimated long-term mean exposure (1995-2005) to THMs in CWS serving participants’ residential addresses, time-weighted by the duration spent at each address.^37^ Figure 1 shows the map of California CWS boundaries and mean THM concentrations.

#### Residential Community Water Supplies

We obtained CWS boundaries from the California Office of Environmental Health Hazard Assessment (OEHHA), previously collected from water system operators and local agencies in a statewide effort.^38,39,40,41,42^ These boundaries were linked to geocoded residential addresses of CTS participants from enrollment to the end of follow-up, which were tracked through the postal service and commercial and credit agencies and self-reported in follow-up surveys.^37^ Validation of CWS assignment in the CTS compared with self-reported water source is described elsewhere, with generally high agreement.^38,39^

#### Trihalomethane Concentrations

CWS routinely measure regulated contaminants, including THMs, and report concentrations to the California Safe Drinking Water Information System.^43^ We used these data to estimate annual mean concentrations of THMs using procedures documented by OEHHA and used in other CTS water analyses.^39,40,41^ We imputed concentrations below the detection limit for reporting using Tobit regression based on existing measurement data and assuming a log-normal distribution, replacing about 30% of samples with values bound by 0 to 0.5 μg/L.^39,44^

Data include annual concentrations of total THMs (TTHM) and 4 individual THMs (chloroform, bromodichloromethane, dibromochloromethane, and bromoform). To improve the completeness of our data, we used an interpolation method replacing missing years with the mean concentration from adjacent years, following the US EPA stage 1 and stage 2 monitoring timelines (eTable 3 in Supplement 1).^38,45^

#### Exposure Assessment

Annual THM concentrations in CWS were linked to participants’ residential addresses, corresponding to the years lived at each address. We evaluated exposure to TTHM, the sum of brominated THMs (bromodichloromethane, dibromochloromethane, and bromoform), chloroform, and individual brominated THMs. The correlation matrix of contaminants is available in eTable 4 in Supplement 1. We calculated residence-weighted mean concentrations from 1995 to 2005 (time between enrollment and the start of follow-up). We also computed the number of years where the TTHM concentration was half or more of the MCL (40 μg/L).

To account for changes in THMs over time and residential mobility over the course of follow-up, we used a dynamic time-varying approach in a secondary analysis, leveraging additional years of data (eTable 3 in Supplement 1).^38^ In this approach, exposure was calculated as a cumulative moving mean since the start of the exposure period (1995), recalculated for each year of follow-up until a participant’s end of follow-up. To account for latency between exposure and CKD diagnosis, we also lagged the cumulative mean by 5 years.

### Statistical Analysis

We used mixed-effects Cox proportional hazards regression models to estimate hazard ratios (HRs) and 95% CIs for CKD risk by THM levels in CWS. We first evaluated mean exposure continuously using flexible cubic restricted splines. Exposures were then categorized by quartiles (Q1 to Q4) and further split at the 95th percentile. To test for exposure-response associations, we calculated *P* value trends by modeling the median of each percentile category. We also examined continuous exposure per IQR.

Age at the start and end of each participant’s follow-up was used as the time variable. We adjusted our models for potential confounders, identified from previous literature and conceptualized using a directed acyclic graph (eFigure 2 in Supplement 1).^46,47,48^ This included self-reported information collected at enrollment: body mass index (BMI), smoking status,^49^ and race and ethnicity. We also present results unadjusted for race and ethnicity. Environmental and health inequities that affect racial and ethnic groups and arise from social factors, including historic redlining and disparities in health care access, can contribute to greater exposure to drinking water contaminants and an increased risk of CKD.^50,51,52,53,54^ We also included quartiles of neighborhood socioeconomic status (SES), derived from 1990 Census block data.^39,55^ The California Census region^56^ (Figure 1) where participants lived at the start of follow-up was included as a random effect to account for geographic differences in CKD incidence, observed in our data,^33^ that likely stem from differences in health care access and nephrology care.^57^ In sensitivity analyses, we further adjusted for other risk factors, including self-reported diabetes, hypertension, menopause status, exercise (hours per week), and smoking pack-years at enrollment. We also conducted a sensitivity analysis using the Fine and Gray subdistribution hazards model to account for death as a potential competing risk.^58^

In secondary analyses, we evaluated differential associations between THM exposure and CKD risk, stratifying and testing for multiplicative interaction by established risk factors, including age, BMI, and smoking status at enrollment. We used g-computation to estimate both the comparative contribution of water contaminants and their joint association (per IQR) with CKD risk, including brominated THMs, chloroform, and mean uranium and arsenic concentrations in residential CWS (1995-2005)—potential nephrotoxic metals previously evaluated in the CTS.^33,59^ We also evaluated the associations between CKD and 5 haloacetic acids, another group of regulated disinfection byproducts, that were available for a limited subset of participants during the exposure period.^39^ Our analyses were conducted using R, version 4.3.3 (R Project for Statistical Computing) in the CTS Researcher Platform.^60^ Statistical analysis was conducted from July 2023 to December 2024. Two-sided *P* < .05 was considered significant.

## Results

Out of 133 477 females enrolled in the CTS, this study included 89 320 with 6242 incident moderate or greater CKD cases. The median age at enrollment was 50 years (IQR, 43-61 years). In all, 3.3% of participants were Asian, 2.8% were Black or African American, 4.8% were Hispanic or Latina, 85.7% were White, and 3.4% were of an additional category or had unknown race and ethnicity. Compared with participants without CKD, those with moderate or greater CKD were more likely to be older than 55 years, to have overweight or obesity, to have ever smoked, to be Black or African American vs all other race and ethnicity categories, and to live in urban areas (eg, Bay Area) rather than rural areas (eg, San Joaquin Valley) (Table 1). In all, 92.0% of participants lived in an area served by a CWS. Median concentrations were 5.5 μg/L (IQR, 0.5-24.1 μg/L) for TTHM, 2.7 μg/L (IQR, 0.7-11.3 μg/L) for brominated THMs, and 2.4 μg/L (IQR, 0.3-9.1 μg/L) for chloroform. THM levels were generally similar across participant characteristics but were somewhat higher among participants who were Black or African American and those living in higher-SES neighborhoods, likely reflecting higher THMs in metropolitan areas.^39^ Exposure was highest in urban coastlines, including San Diego (median TTHM concentration, 41.9 μg/L [IQR, 1.1-51.8 μg/L]) and the Bay Area (24.1 μg/L [IQR, 1.9-57.8 μg/L]).

**Table 1.** Participant Characteristics by Chronic Kidney Disease Status and THM Exposure^a^

Characteristic	**Participants (N = 89 320)**				
	CKD absent, No. (%)	CKD present, No. (%)	Total THM, median (IQR), μg/L	Brominated THM, median (IQR), μg/L	Chloroform, median (IQR), μg/L
Total	83 078 (93.0)	6242 (7.0)	5.5 (0.5-24.1)	2.7 (0.7-11.3)	2.4 (0.3-9.1)
Age, y					
22-34	9748 (11.7)	51 (0.8)	5.6 (0.7-20.1)	2.8 (0.8-10.8)	2.3 (0.4-8.2)
35-44	16 674 (20.1)	225 (3.6)	5.2 (0.4-21.4)	2.4 (0.6-10.8)	2.2 (0.3-8.4)
45-54	27 230 (32.8)	1239 (19.8)	5.4 (0.5-25.5)	2.7 (0.7-11.6)	2.5 (0.3-9.5)
55-64	15 259 (18.4)	2008 (32.2)	5.7 (0.6-25.5)	2.8 (0.7-11.6)	2.6 (0.3-9.5)
65-85	14 167 (17.1)	2719 (43.6)	6.1 (0.5-24.2)	2.9 (0.7-11.6)	2.6 (0.3-9.5)
BMI^b^					
Underweight, <18.5	2252 (2.7)	92 (1.5)	6.2 (0.5-25.5)	2.9 (0.7-11.6)	2.6 (0.3-9.5)
Normal weight, 18.5-24.9	49 606 (59.7)	2500 (40.1)	5.8 (0.5-24.4)	2.8 (0.7-11.6)	2.5 (0.3-9.5)
Overweight, 25.0-29.9	20 244 (24.4)	2000 (32.0)	5.3 (0.5-23.5)	2.6 (0.7-10.9)	2.4 (0.3-8.9)
Obese, ≥30.0	10 976 (13.2)	1650 (26.4)	5.2 (0.5-20.1)	2.4 (0.7-10.6)	2.2 (0.3-7.9)
Smoking status					
Never smoker	16 610 (20.0)	867 (13.9)	5.2 (0.5-23.3)	2.4 (0.6-10.9)	2.2 (0.3-8.8)
Never smoker, household exposure^c^	39 257 (47.3)	2812 (45.0)	5.3 (0.5-23.3)	2.5 (0.7-10.9)	2.3 (0.3-8.9)
Former smoker	23 314 (28.1)	2175 (34.8)	7.3 (0.7-25.5)	3.0 (0.8-11.6)	2.8 (0.3-9.5)
Current smoker	3897 (4.7)	388 (6.2)	5.4 (0.5-21.4)	2.8 (0.7-10.8)	2.5 (0.3-8.7)
Race and ethnicity					
Asian	2869 (3.5)	123 (2.0)	5.2 (0.2-22.8)	2.5 (0.5-9.6)	1.8 (0.2-7.7)
Black or African American	2188 (2.6)	323 (5.2)	9.2 (0.6-17.5)	5.2 (1.6-10.8)	3.8 (0.6-7.0)
Hispanic or Latina	4071 (4.9)	175 (2.8)	3.6 (0.3-17.2)	2.3 (0.6-10.8)	1.6 (0.2-6.7)
White	71 144 (85.6)	5421 (86.8)	5.7 (0.6-24.1)	2.7 (0.7-11.6)	2.5 (0.3-9.5)
Additional categories or unknown^d^	2806 (3.4)	200 (3.2)	5.3 (0.4-21.8)	2.8 (0.7-10.8)	2.4 (0.3-8.2)
Neighborhood SES quartile^e^					
1	2859 (3.4)	213 (3.4)	1.8 (0.3-15.3)	1.2 (0.3-5.1)	1.0 (0.1-7.0)
2	12 491 (15.0)	964 (15.4)	3.6 (0.3-16.2)	1.7 (0.4-7.0)	1.7 (0.2-7.0)
3	27 265 (32.8)	1989 (31.9)	5.2 (0.4-18.3)	2.3 (0.6-10.4)	2.2 (0.3-8.2)
4 (Most affluent)	40 463 (48.7)	3076 (49.3)	8.7 (0.8-27.7)	4.3 (0.9-13.8)	3.4 (0.4-9.5)
California census region^f^					
San Diego	8507 (10.2)	608 (9.7)	41.9 (1.1-51.8)	28.6 (2.8-35.4)	12.5 (1.3-16.9)
Bay Area	15 489 (18.6)	1466 (23.5)	24.1 (1.9-57.8)	9.5 (1.1-15.5)	7.3 (1.0-53.6)
North Coast	2576 (3.1)	191 (3.1)	11.4 (5.3-15.3)	4.9 (2.8-8.8)	2.5 (1.3-9.1)
Central Coast	6813 (8.2)	336 (5.4)	8.8 (1.4-24.7)	3.7 (1.3-14.2)	3.7 (0.6-5.6)
Superior	7236 (8.7)	553 (8.9)	7.8 (0.7-12.6)	0.8 (0.4-1.3)	6.5 (0.4-10.3)
Los Angeles County	17 829 (21.5)	1514 (24.3)	5.2 (0.3-17.2)	5.2 (1.1-11.6)	2.2 (0.5-5.8)
North San Joaquin	3193 (3.8)	164 (2.6)	4.7 (0.1-26.7)	1.1 (0.2-1.7)	3.7 (0.1-20.6)
Inland Empire	7789 (9.4)	534 (8.6)	1.9 (0.6-7.6)	2.1 (0.6-5.1)	0.9 (0.1-3.2)
Orange County	8714 (10.5)	635 (10.2)	1.2 (0.8-8.4)	1.0 (0.7-3.7)	0.4 (0.3-1.8)
South San Joaquin	4932 (5.9)	241 (3.9)	0.2 (0.2-2.0)	0.3 (0.3-0.8)	0.1 (0.1-1.7)

Abbreviations: BMI, body mass index (calculated as weight in kilograms divided by height in meters squared); CKD, chronic kidney disease; THM, trihalomethane.

^a^ Mean total THM, brominated THM, and chloroform concentrations in community water supplies serving residential addresses from 1995 to 2005, stratified by characteristics of California Teachers Study participants at enrollment (N = 89 320), by incident moderate or greater chronic kidney disease status.

^b^ For 1476 participants, self-reported BMI from the fourth questionnaire (2005-2007) was used in place of information missing from the first questionnaire.

^c^ Defined as ever living with a parent or adult that smoked in the home.

^d^ Due to limited numbers, we consolidated American Indian or Alaska Native (731 participants [0.8%]), Native Hawaiian or Other Pacific Islander (569 [0.6%]), multiracial (975 [1.1%]), or not reported (732 [0.8%]) but recognize that each of these groups is unique and does not share a common identity.

^e^ Summary socioeconomic status is a metric of occupation, educational level, and income at the census-block level (1990), categorized into quartiles across California. Estimated from the enrollment address.

^f^ Region in which participants resided at the start of follow-up in 2005.

**Figure 2.**
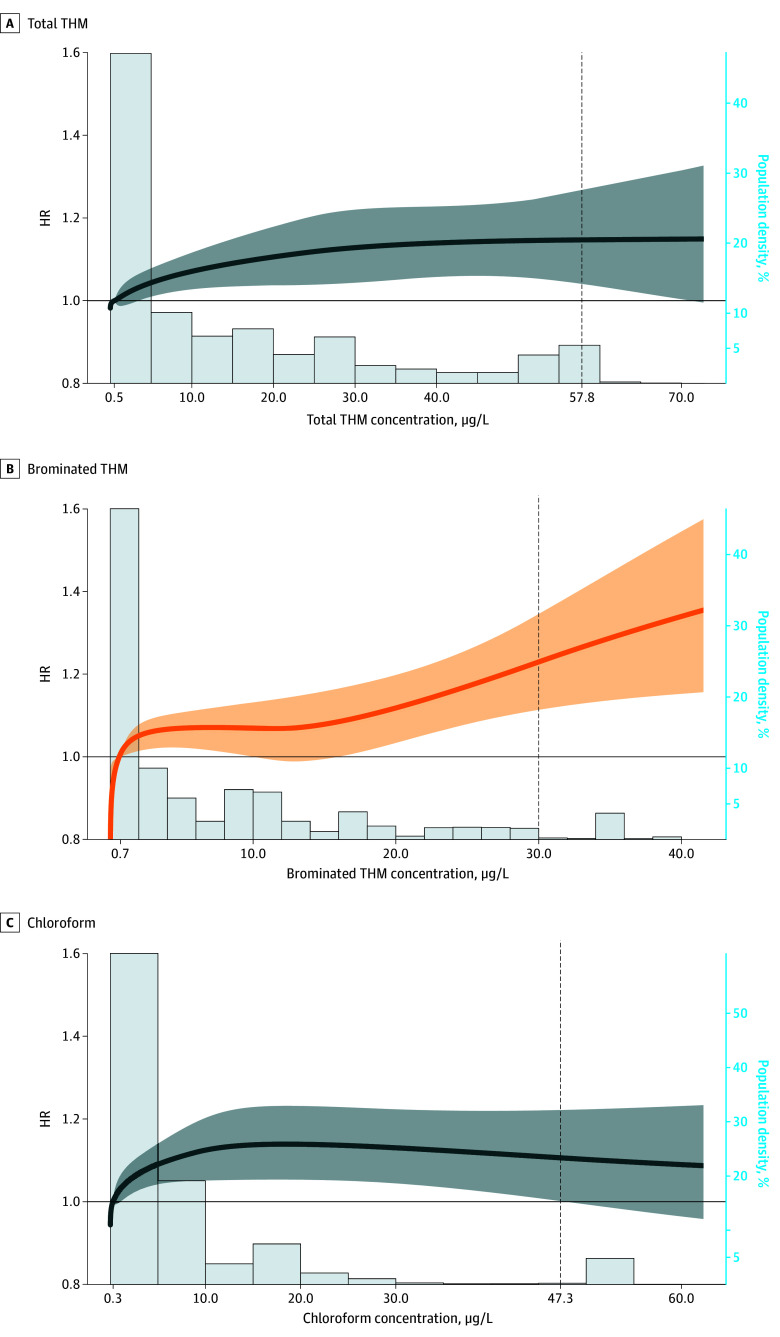
Exposure-Response Associations Between Trihalomethane (THM) Concentrations in Residential Community Water Supplies and Moderate to End-Stage Chronic Kidney Disease (1995-2005) Solid lines indicate hazard ratios (HRs) for chronic kidney disease and shading, 95% CIs, modeled as restricted cubic splines in the California Teachers Study (N = 89 320; analytic follow-up, 2005-2018). HRs were adjusted for body mass index as a continuous variable, smoking status, race and ethnicity, neighborhood socioeconomic status quartiles, US Census region as a random effect, and age as the time scale. Concentrations were log-transformed with knots placed at the 25th, 75th, and 95th percentiles. Vertical dashed lines indicate the 95th percentiles and the lowest concentration in each graph indicates the 25th percentile. A histogram of the population density is shown for each spline. To better visualize trends at the lower tails, plots with x-axes on a log scale are provided in eFigure 3 in Supplement 1.

Using restricted cubic splines, we observed evidence of a positive exposure-response association between TTHM and moderate or greater CKD risk (hazard ratio [HR], 1.15 [95% CI, 1.04-1.26] at 57.8 μg/L [95th percentile] vs 0.5 μg/L [25th percentile]) (Figure 2 and eFigure 3 in Supplement 1). The HR was higher for brominated THMs (1.22 [95% CI, 1.11-1.35] at 30.0 μg/L [95th percentile] vs 0.7 μg/L [25th percentile]) and markedly lower for chloroform (1.11 [95% CI, 1.00-1.22] at 47.3 μg/L [95th percentile] vs 0.3 μg/L [25th percentile]). Brominated THM exposure was associated with CKD risk across percentile categories (*P* < .001 for trend). In comparison with Q1, the HRs for brominated THMs at the highest exposure categories (Q4 and ≥95th percentile) were 1.23 (95% CI, 1.13-1.33) and 1.43 (95% CI, 1.23-1.66), respectively (Table 2). The corresponding HRs for TTHM were 1.16 (95% CI, 1.07-1.26) and 1.14 (95% CI, 1.01-1.28), respectively. Among individual THMs, the HR for dibromochloromethane for the 95th percentile vs the median was 1.33 (95% CI, 1.15-1.54); for bromodichloromethane, the HR for the 95th percentile vs Q1 was 1.32 (95% CI, 1.13-1.54) and for Q4 vs Q1 was 1.15 (95% CI, 1.06-1.24); and for chloroform, the HR for the 95th percentile vs Q1 was 1.12 (95% CI, 0.99-1.26), with no significant exposure-response trend (*P* = .11 for trend). From a regulatory standpoint, participants exposed to TTHM concentrations that were half or more of the MCL (40 μg/L) for 4 or more years had an increased risk of CKD (HR, 1.11 [95% CI, 1.02-1.20]) compared with those who had no years of exposure at this threshold.

**Table 2.** CKD Risk Associated With Mean Concentrations of Total, Brominated, and Individual Trihalomethanes From Residential Community Water Supplies in the California Teachers Study

	Participants, No.	Moderate or greater CKD^a^			Advanced or end-stage CKD^b^		All stages of CKD^c^	
		Cases, No.	HR (95% CI)^d^	Without race and ethnicity data, HR (95% CI)^e^	Cases, No.	HR (95% CI)^d^	Cases, No.	HR (95% CI)^d^
**Total trihalomethanes, μg/L**								
Q1 (≤0.4)	21 144	1355	1 [Reference]	1 [Reference]	344	1 [Reference]	2032	1 [Reference]
Q2 (0.5-5.4)	23 550	1567	1.03 (0.96-1.11)	1.04 (0.96-1.12)	365	0.95 (0.82-1.10)	2247	1.00 (0.94-1.06)
Q3 (5.5-24.0)	22 256	1567	1.09 (1.01-1.18)	1.10 (1.02-1.19)	394	1.08 (0.94-1.26)	2189	1.02 (0.96-1.09)
Q4 (24.1-57.7)	17 592	1303	1.16 (1.07-1.26)	1.16 (1.07-1.26)	303	1.11 (0.95-1.30)	1796	1.10 (1.02-1.17)
≥95th percentile (57.8-93.7)	4778	450	1.14 (1.01-1.28)	1.17 (1.04-1.32)	99	1.13 (0.89-1.43)	582	1.02 (0.93-1.13)
*P* value for trend^f^	NA	NA	<.001	<.001	NA	.04	NA	.04
Continuous (IQR)^g^	NA	NA	1.07 (1.03-1.11)	1.07 (1.04-1.11)	NA	1.08 (1.01-1.16)	NA	1.04 (1.01-1.07)
Time exposed to ≥½ MCL (40 μg/L), y^h^								
0	66 059	4568	1 [Reference]	1 [Reference]	1106	1 [Reference]	6558	1 [Reference]
1-3	10 117	632	1.09 (1.00-1.19)	1.10 (1.01-1.20)	156	1.04 (0.88-1.23)	886	1.05 (0.97-1.12)
4-11	13 144	1042	1.11 (1.02-1.20)	1.12 (1.04-1.21)	243	1.14 (0.98-1.32)	1402	1.07 (1.00-1.14)
**Brominated trihalomethanes, μg/L**								
Q1 (≤0.6)	21 569	1319	1 [Reference]	1 [Reference]	322	1 [Reference]	1981	1 [Reference]
Q2 (0.7-2.6)	22 892	1603	1.17 (1.08-1.26)	1.18 (1.09-1.27)	369	1.10 (0.95-1.28)	2271	1.12 (1.06-1.20)
Q3 (2.7-11.2)	22 477	1593	1.08 (1.00-1.17)	1.10 (1.02-1.19)	416	1.20 (1.03-1.39)	2250	1.03 (0.97-1.10)
Q4 (11.3-29.9)	17 783	1387	1.23 (1.13-1.33)	1.22 (1.13-1.32)	311	1.17 (1.00-1.37)	1895	1.13 (1.06-1.21)
≥95th percentile (30.0-42.9)	4599	340	1.43 (1.23-1.66)	1.44 (1.24-1.68)	87	1.46 (1.14-1.88)	449	1.30 (1.14-1.47)
*P* value for trend^f^	NA	NA	<.001	<.001	NA	.006	NA	<.001
Continuous (IQR)^g^	NA	NA	1.08 (1.04-1.11)	1.07 (1.04-1.11)	NA	1.09 (1.03-1.16)	NA	1.05 (1.02-1.08)
**Individual trihalomethanes, μg/L**								
Chloroform								
Q1 (≤0.2)	20 382	1279	1 [Reference]	1 [Reference]	309	1 [Reference]	1923	1 [Reference]
Q2 (0.3-2.3)	23 767	1595	1.06 (0.98-1.14)	1.06 (0.98-1.15)	400	1.12 (0.96-1.30)	2303	1.04 (0.98-1.11)
Q3 (2.4-9.0)	22 821	1698	1.14 (1.06-1.23)	1.16 (1.07-1.25)	427	1.18 (1.02-1.37)	2326	1.05 (0.99-1.12)
Q4 (9.1-47.2)	17 866	1243	1.12 (1.03-1.22)	1.12 (1.03-1.22)	272	1.05 (0.89-1.24)	1737	1.06 (0.99-1.14)
≥95th percentile (47.3-84.1)	4484	427	1.12 (0.99-1.26)	1.15 (1.02-1.30)	97	1.23 (0.98-1.56)	557	1.02 (0.92-1.13)
*P* value for trend^f^	NA	NA	.11	.05	NA	.35	NA	.75
Continuous (IQR)^g^	NA	NA	1.02 (1.00-1.04)	1.02 (1.00-1.04)	NA	1.02 (0.98-1.05)	NA	1.01 (0.99-1.02)
Bromodichloromethane								
Q1 (≤0.2)	21 694	1378	1 [Reference]	1 [Reference]	327	1 [Reference]	2069	1 [Reference]
Q2 (0.3-1.3)	22 920	1573	1.06 (0.99-1.15)	1.08 (1.00-1.16)	380	1.12 (0.97-1.30)	2216	1.03 (0.96-1.09)
Q3 (1.4-5.7)	22 226	1539	1.06 (0.98-1.14)	1.07 (0.99-1.16)	382	1.12 (0.96-1.30)	2201	1.01 (0.95-1.08)
Q4 (5.8-16.3)	18 004	1422	1.15 (1.06-1.24)	1.15 (1.06-1.25)	333	1.23 (1.06-1.44)	1927	1.07 (1.00-1.14)
≥95th percentile (16.4-21.1)	4476	330	1.32 (1.13-1.54)	1.34 (1.15-1.56)	83	1.39 (1.09-1.78)	433	1.19 (1.04-1.36)
*P* value for trend^f^	NA	NA	<.001	<.001	NA	.003	NA	.006
Continuous (IQR)^g^	NA	NA	1.07 (1.04-1.11)	1.08 (1.04-1.11)	NA	1.09 (1.03-1.15)	NA	1.04 (1.02-1.07)
Dibromochloromethane^i^								
Q1-2 (≤0.7)	43 081	2816	1 [Reference]	1 [Reference]	665	1 [Reference]	4099	1 [Reference]
Q3 (0.8-4.5)	23 897	1718	1.03 (0.96-1.10)	1.05 (0.98-1.12)	443	1.13 (1.00-1.29)	2428	1.00 (0.95-1.06)
Q4 (4.6-12.0)	17 828	1377	1.12 (1.04-1.20)	1.12 (1.04-1.20)	310	1.12 (0.97-1.29)	1880	1.06 (1.00-1.12)
≥95th percentile (12.1-18.3)	4514	331	1.33 (1.15-1.54)	1.35 (1.17-1.57)	87	1.45 (1.14-1.85)	439	1.24 (1.09-1.40)
*P* value for trend^f^	NA	NA	<.001	<.001	NA	.006	NA	<.001
Continuous (IQR)^g^	NA	NA	1.08 (1.04-1.11)	1.07 (1.04-1.11)	NA	1.10 (1.04-1.16)	NA	1.05 (1.02-1.08)
Bromoform^i^								
Q1-2 (≤0.1)	40 038	2740	1 [Reference]	1 [Reference]	630	1 [Reference]	3932	1 [Reference]
Q3 (0.2-1.4)	25 576	1698	1.03 (0.96-1.09)	1.04 (0.98-1.11)	440	1.09 (0.96-1.23)	2444	1.01 (0.96-1.07)
Q4 (1.5-3.0)	19 084	1468	1.09 (1.02-1.17)	1.08 (1.00-1.16)	356	1.19 (1.04-1.36)	2008	1.04 (0.98-1.10)
≥95th percentile (3.1-18.1)	4622	336	1.23 (1.10-1.39)	1.22 (1.09-1.38)	79	1.23 (0.97-1.56)	462	1.19 (1.08-1.32)
*P* value for trend^f^	NA	NA	<.001	<.001	NA	.011	NA	.003
Continuous (IQR)^g^	NA	NA	1.07 (1.03-1.10)	1.06 (1.03-1.10)	NA	1.09 (1.02-1.16)	NA	1.04 (1.01-1.07)

Abbreviations: CKD, chronic kidney disease; HR, hazard ratio; MCL, maximum contaminant level; NA, not applicable.

^a^ Includes CKD stages 3 to 5 and end-stage kidney disease.

^b^ Includes CKD stages 4 to 5 and end-stage kidney disease.

^c^ Includes CKD stages 1 to 5 and end-stage kidney disease.

^d^ Adjusted for body mass index as a continuous variable, smoking status, race and ethnicity, neighborhood socioeconomic status quartiles, census region as a random effect, and age as the time scale.

^e^ Race and ethnicity were removed from the model to evaluate the associations in the context that exposures are unequally distributed and can have differential consequences for populations due to discrimination and the presence of additional environmental stressors.

^f^ Modeled as a continuous variable derived from the median of each exposure category.

^g^ Concentrations were modeled continuously, and HRs are expressed per IQR.

^h^ Categorized by 0 years of exposure and further split by less than or greater than or equal to the median years for those with at least 1 year of exposure at or above one-half the MCL threshold.

^i^ Values below the median level were used as the reference category because of the limited concentration distribution.

Compared with moderate or greater CKD, associations with advanced or end-stage CKD were similar to and somewhat attenuated for all CKD stages (Table 2). The HRs were somewhat higher when not adjusted for race and ethnicity and were similar when further adjusted for other potential confounders (eTable 5 in Supplement 1). Results remained consistent when accounting for death as a competing risk (eTable 6 in Supplement 1). In time-varying analyses, there was also an association between cumulative moving mean TTHM exposure and CKD risk, with higher HRs than in our main analysis (HR for the 95th percentile vs Q1, 1.37; 95% CI, 1.21-1.56) (eTable 7 in Supplement 1). CKD risk from brominated THMs was greater at lower percentiles than in our main analysis, especially when lagged by 5 years, with an HR of 1.36 (95% CI, 1.26-1.47) at Q2 and 1.32 (95% CI, 122-1.44) at Q3. We found no statistically significant interactions between THM exposure and participant characteristics (eTable 8 in Supplement 1).

**Figure 3.**
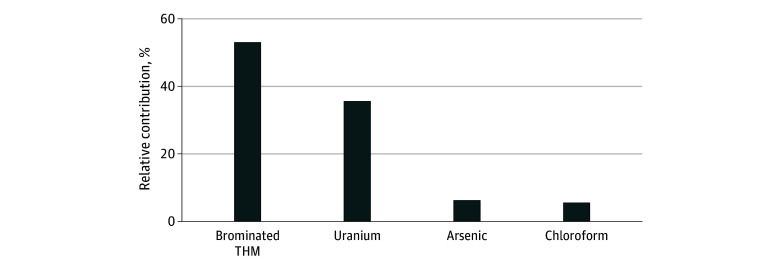
Relative Contributions of Water Contaminants to Chronic Kidney Disease (CKD) Risk Mixture analysis used g-computation to estimate the relative contributions of mean brominated trihalomethane (THM), chloroform (nonbrominated), uranium, and arsenic concentrations from residential community water supplies (1995-2005) to the overall association with moderate to end-stage chronic kidney disease (CKD) risk in the California Teachers Study (analytic follow-up, 2005-2018). Analysis included 88 169 participants with complete uranium and arsenic data (all but 1151 of the total 89 320 participants). The overall outcome (hazard ratio and 95% CI) was the risk of CKD associated with a joint IQR increase of all water contaminants. Findings were adjusted for body mass index as a continuous variable, smoking status, race and ethnicity, neighborhood socioeconomic status quartiles, census region as a stratification term, and age as the time scale.

Correlations between individual THMs were high, especially among brominated THMs (Spearman ρ = 0.68-0.91) (eTable 4 in Supplement 1). In our mixture analysis including brominated THMs, chloroform, uranium, and arsenic exposure, the joint increase of all contaminants per IQR was associated with an increased risk of CKD (HR, 1.15; 95% CI, 1.08-1.21) (Figure 3). Brominated THMs were the largest contributor (52.9%) to the overall association with CKD, while chloroform had the smallest influence (5.5%). Uranium was the second largest contributor (35.4%), while the contribution of arsenic (6.2%) was similar to chloroform. Data on haloacetic acids were available for 32 277 participants (36.1%) during the exposure period; no associations were found between haloacetic acids and CKD (eTable 9 in Supplement 1).

## Discussion

In a large study of female educators from California, decade-long exposure to higher levels of trihalomethanes in drinking water was associated with a higher risk of subsequent CKD development. TTHM exposure in this study population was well below the regulatory limit (≤80 μg/L), suggesting that current policy may not protect against long-term risk. In our study, brominated THMs were associated with the greatest CKD risk and emerged as the largest contributor in our mixture analysis. Brominated THMs are not separately regulated from TTHM, although prior evidence suggests that they may be more nephrotoxic than chloroform.^7,23^ These novel findings require further investigation, especially in the broader context of the global CKD burden, widespread chlorination practices, and water quality threats from climate change.

Our findings are supported by toxicologic evidence that brominated compounds are especially toxic to the kidney and other organ systems.^29,30,31^ Animal studies provide considerable evidence of kidney toxic effects from brominated THMs (particularly bromodichloromethane), including proximal tubular damage and reduced GFR.^24,25,26,27,28,30^ Brominated THMs, unlike chloroform, have been shown to be mutagenic when metabolized through glutathione S-transferase theta 1,^61^ an important detoxifying enzyme abundant in the kidneys.^62^ In addition, brominated compounds may interfere with bromine enrichment in the glomerular basement membrane, an essential element in collagen scaffold assembly and structural integrity.^63,64^ Furthermore, THM exposure has been extensively associated with intrauterine growth restriction (IUGR),^21,22^ which is associated with CKD development later in life.^65,66,67^ Continued exposure to THMs among individuals with IUGR may exacerbate risk and warrants further investigation.

To our knowledge, there are no longitudinal epidemiologic studies evaluating trihalomethanes from drinking water and CKD risk to directly compare with these findings. Our findings are generally consistent with cross-sectional results from NHANES, where higher blood levels of bromodichloromethane and chloroform were associated with lower estimated GFR and dibromochloromethane was associated with a higher urinary albumin-to-creatinine ratio.^32^ Another NHANES study (1999-2018) showed an association between brominated THMs and hypertension—a frequent comorbidity associated with CKD.^68^

California’s OEHHA has proposed noncancer public health goals for each of the 4 THMs, with the lowest limit set at 13 μg/L for bromodichloromethane.^23^ Chronic exposure to bromodichloromethane within the range of the recommended limit (5.8-16.3 μg/L; Q4) was associated with a 15% increased risk of CKD in comparison with 0.2 μg/L or less (Q1), while concentrations at 16.4 to 21.1 μg/L (≥95th percentile) were associated with a 32% increased risk. While the high correlation of the individual brominated THMs limited our ability to discern their comparative contributions to CKD risk, we found that the contribution of total brominated compounds to CKD risk was substantially larger (52.9%) than chloroform (5.5%), the only nonbrominated compound in the TTHM. Brominated THMs often occur at lower concentrations than chloroform, as observed in our data. While CWS may meet the current standard for TTHM, water systems with a higher proportion of brominated compounds could pose greater risk to health, suggesting the possible need for greater monitoring and specific regulation for brominated THMs.

Our study included extensive water quality data linked to participants’ residential addresses since enrollment, including residential mobility within California.^37,39^ These data allowed us to estimate mean THM exposure for about a decade, weighted by residence and time spent at each address. We further leveraged THM data available for additional years using a time-varying approach to estimate cumulative mean exposure, consistently observing significant exposure-response associations, including at lower levels of brominated THM exposure. These are notable strengths to our study, as most environmental studies often rely only on the baseline address. Studies that measure THMs in biospecimens often rely on a single sample and therefore do not capture changes in water THM exposure over time, and they are further limited by the very short half-lives of THMs (about 4 hours).^69^

This study adds to the growing body of literature describing the role of environmental exposures in CKD.^5,6^ In our mixture analysis that evaluated a number of drinking water contaminants simultaneously, we found that brominated THMs and uranium were the largest contributors to overall CKD risk, which is consistent with a previous study by our group.^33^ However, it should be noted that uranium and THMs are unlikely to co-occur (Spearman ρ = −0.14) (eTable 4 in Supplement 1), supporting that these are independent CKD risk factors. Several other environmental factors have also been associated with CKD risk, including air pollution, pesticides, heat, and dehydration, underscoring the need to examine the compounding risk of multiple environmental insults.^70^ For example, THMs may affect urinary concentrating ability, which could impair volume regulation and exacerbate recurrent acute kidney injury in hot climates.^29,71^ These findings may even be relevant to CKD of uncertain origin, which is not explained by typical risk factors (eg, hypertension, obesity, or diabetes) and is marked by significant tubular injury.^71,72^ California and many other places in the world face several climate change challenges, including rising temperatures, coastal flooding, acidification, and organic matter from pollution and wildfires, which are likely to exacerbate THM formation in chlorinated water.^14,15,16,17,18,73^ This is particularly relevant to San Diego and the Bay Area, densely populated coastal regions where we observed the highest THM exposures.

### Limitations

This study has limitations. We could not account for individual behaviors influencing THM exposure, including daily water intake, tap water and water filtration use, dermal exposure during bathing or showering, or the use of water sources outside the house.^74,75^ However, residential tap water THMs are a major source of exposure and correlate with blood THM concentrations, which reflect exposure from all sources (Spearman ρ for brominated THMs = 0.54-0.62).^69,76,77^ Tap water THM concentrations have been shown to be more predictive of exposure than individual water use habits.^76,77^ There were limited measurement data on haloacetic acids, another group of disinfection byproducts that often co-occur with THMs, with some evidence of nephrotoxicity.^78^ While other disinfection byproducts may contribute to CKD risk, THMs have been shown to independently impair kidney function in controlled animal studies.^24,25,26^ Another limitation of our work was the inability to assess exposures at nonresidential locations, such as workplaces, since employment addresses were not collected in the cohort.

Our study was also limited by the use of administrative data, where CKD may be underrecognized. However, our study’s ascertainment of cases for over 10 years helps mitigate this limitation by improving case capture and sensitivity over time.^79^ Given the study period, CKD diagnoses were likely based on older estimated GFR equations that included race as a coefficient, which may have introduced misclassification.^80^ Associations were present for moderate and advanced CKD and were attenuated when including all stages, which may suggest greater misclassification in earlier stages, particularly stage 1. Our study lacked biomarkers of kidney injury, including albuminuria, proteinuria, and other markers of tubular injury, limiting our ability to investigate underlying mechanisms, which should be prioritized in future research to better elucidate our results. The study’s population of female public school teachers limits the generalizability of our findings. However, the CTS is a well-established cohort of substantial size, with extensive follow-up, health outcome ascertainment, and residential address tracking since 1995, enabling researchers to conduct large-scale environmental exposure studies like the current study. Our findings warrant further investigation in other settings and study designs, including in cohorts with greater diversity and biomarker data.

## Conclusions

In this prospective cohort study of California female teachers, we observed a clear exposure-response association between trihalomethane concentrations in residential water and CKD risk. Brominated compounds, which are not separately regulated, emerged as the primary contributors to CKD risk, with associations observed at levels below the current US regulatory limit for community water supplies. These findings are supported by substantial experimental evidence and carry important public health implications given the widespread use of water chlorination and the growing global burden of CKD.^4,9^

## Notes

Supplement 1. **eTable 1.** Coding Procedures for the Original Race and Ethnicity Categories Reported at Enrollment, in Compliance With National Institutes of Health Reporting Requirements **eTable 2.** Definitions of Chronic Kidney Disease and Comorbidities in the California Teachers Study From Administrative Records **eTable 3.** Trihalomethane Data From Community Water Supplies Serving the Residential Addresses of California Teachers Study Participants **eTable 4.** Correlation Between Total, Brominated, and Individual Trihalomethanes, Uranium and Arsenic, and Haloacetic Acid Concentrations **eTable 5.** Further Adjustments of Hazard Ratios and 95% CIs for Chronic Kidney Disease Risk Associated With Concentrations of Total, Brominated, and Individual Trihalomethanes From Residential Community Water Supplies **eTable 6.** Competing Risk Models Including Death as a Competing Risk for Chronic Kidney Disease **eTable 7.** Time-Varying Analyses of Cumulative Moving Average Total and Brominated Trihalomethane Exposure From Residential Community Water Supplies Since 1995 **eTable 8.** Stratified Analyses by Baseline Age, BMI, and Smoking Status in the California Teachers Study **eTable 9.** Hazard Ratios and 95% CIs for Chronic Kidney Disease Risk Associated With Concentrations of Haloacetic Acids From Residential Community Water Supplies in the California Teachers Study **eFigure 1.** Inclusion Flowchart for Analysis of Trihalomethane Exposure From Community Water Supplies and Chronic Kidney Disease Risk in the California Teachers Study **eFigure 2.** Directed Acyclic Graph Conceptualizing Potential Confounding Factors Between Trihalomethane Exposure From Community Water Supplies and the Development of Chronic Kidney Disease **eFigure 3.** Hazard Ratios and 95% CIs for Moderate or Greater Chronic Kidney Disease Risk, With X-Axis on a Log Scale and Labels at the 10th, 25th, 50th, 75th, and 95th Percentiles

Supplement 2.
Data Sharing Statement
