# Supplementary material for: Community Water Trihalomethanes and Chronic Kidney Disease
Source: JAMA Netw Open. 2025 Jul 2;8(7):e2518513. doi: 10.1001/jamanetworkopen.2025.18513 (PMC12223891; doi:10.1001/jamanetworkopen.2025.18513)
Supplement: Supplement 1. — eTable 1. Coding Procedures for the Original Race and Ethnicity Categories Reported at Enrollment, in Compliance With National Institutes of Health Reporting Requirements eTable 2. Definitions of Chronic Kidney Disease and Comorbidities in the California Teachers Study From Administrative Records eTable 3. Trihalomethane Data From Community Water Supplies Serving the Residential Addresses of California Teachers Study Participants eTable 4. Correlation Between Total, Brominated, and Individual Trihalomethanes, Uranium and Arsenic, and Haloacetic Acid Concentrations eTable 5. Further Adjustments of Hazard Ratios and 95% CIs for Chronic Kidney Disease Risk Associated With Concentrations of Total, Brominated, and Individual Trihalomethanes From Residential Community Water Supplies eTable 6. Competing Risk Models Including Death as a Competing Risk for Chronic Kidney Disease eTable 7. Time-Varying Analyses of Cumulative Moving Average Total and Brominated Trihalomethane Exposure From Residential Community Water Supplies Since 1995 eTable 8. Stratified Analyses by Baseline Age, BMI, and Smoking Status in the California Teachers Study eTable 9. Hazard Ratios and 95% CIs for Chronic Kidney Disease Risk Associated With Concentrations of Haloacetic Acids From Residential Community Water Supplies in the California Teachers Study eFigure 1. Inclusion Flowchart for Analysis of Trihalomethane Exposure From Community Water Supplies and Chronic Kidney Disease Risk in the California Teachers Study eFigure 2. Directed Acyclic Graph Conceptualizing Potential Confounding Factors Between Trihalomethane Exposure From Community Water Supplies and the Development of Chronic Kidney Disease eFigure 3. Hazard Ratios and 95% CIs for Moderate or Greater Chronic Kidney Disease Risk, With X-Axis on a Log Scale and Labels at the 10th, 25th, 50th, 75th, and 95th Percentiles [file jamanetwopen-e2518513-s001.pdf]

Supplementary Online Content

Medgyesi DN, Mohan S, Bangia K, et al. Community water trihalomethanes and chronic kidney disease. *JAMA Netw Open*. 2025;8(7):e2518513. doi:10.1001/jamanetworkopen.2025.18513

eTable 1. Coding Procedures for the Original Race and Ethnicity Categories Reported at Enrollment, in Compliance With National Institutes of Health Reporting Requirements

eTable 2. Definitions of Chronic Kidney Disease and Comorbidities in the California Teachers Study From Administrative Records

eTable 3. Trihalomethane Data From Community Water Supplies Serving the Residential Addresses of California Teachers Study Participants

eTable 4. Correlation Between Total, Brominated, and Individual Trihalomethanes, Uranium and Arsenic, and Haloacetic Acid Concentrations

eTable 5. Further Adjustments of Hazard Ratios and 95% CIs for Chronic Kidney Disease Risk Associated With Concentrations of Total, Brominated, and Individual Trihalomethanes From Residential Community Water Supplies

eTable 6. Competing Risk Models Including Death as a Competing Risk for Chronic Kidney Disease

eTable 7. Time-Varying Analyses of Cumulative Moving Average Total and Brominated Trihalomethane Exposure From Residential Community Water Supplies Since 1995

eTable 8. Stratified Analyses by Baseline Age, BMI, and Smoking Status in the California Teachers Study

eTable 9. Hazard Ratios and 95% CIs for Chronic Kidney Disease Risk Associated With Concentrations of Haloacetic Acids From Residential Community Water Supplies in the California Teachers Study

eFigure 1. Inclusion Flowchart for Analysis of Trihalomethane Exposure From Community Water Supplies and Chronic Kidney Disease Risk in the California Teachers Study

eFigure 2. Directed Acyclic Graph Conceptualizing Potential Confounding Factors Between Trihalomethane Exposure From Community Water Supplies and the Development of Chronic Kidney Disease

eFigure 3. Hazard Ratios and 95% CIs for Moderate or Greater Chronic Kidney Disease Risk, With x-Axis on a Log Scale and Labels at the 10th, 25th, 50th, 75th, and 95th Percentiles

This supplementary material has been provided by the authors to give readers additional information about their work.

© 2025 Medgyesi DN et al. *JAMA Network Open*.

eTable 1. Coding procedures for the original race and ethnicity categories reported at enrollment (1995-1996), in compliance with to the National Institutes of Health reporting requirements.

Value	Description
1 American Indian or Alaskan Native	Native American only or White and Native American reported
2 Asian	Asian only (Chinese, Filipino, Japanese, Vietnamese or Korean) or White and Asian reported
3 Black or African American	Black only or White and Black reported
4 Hispanic or Latino	Hispanic only or White and Hispanic reported
5 Native Hawaiian or Other Pacific Islander	Hawaiian only or White and Hawaiian reported
6 White	White only reported
7 Other or More than one race	Other reported or more than one of the above groups reported

If WHITE and any other race, then the RACE_X variable was coded to the other race. If not WHITE and more than one race was reported then the RACE_X variable was coded to OTHER/MORE THAN ONE RACE.

© 2025 Medgyesi DN et al. *JAMA Network Open*.

eTable 2. Definitions for chronic kidney disease (CKD) and comorbidities in the California Teachers Study from administrative records (2005-2018).

	**Hospitalization data**<br>Patient discharge, emergency department visits and ambulatory surgeries		**Mortality data**<br>Primary underlying cause of death	
	**ICD-9**	**ICD-10 effective October 1, 2015**	**ICD-9**	**ICD-10***
**CKD moderate+ (stage 3-ESRD)**	585.3 CKD stage 3<br>585.4 CKD stage 4<br>585.5 CKD stage 5<br>585.6 ESRD<br>**Procedure codes**<br>447.0 AV fistula<br>996.1 Mechanical complication of other vascular device, implant, and graft<br>7925 Cloudy (hemodialysis) peritoneal dialysis effluent<br>V45.11 Renal Dialysis status<br>V42.0 Kidney replaced by transplant<br>V56.0 Encounter for extracorporeal dialysis<br>V45.12 Noncompliance with renal dialysis<br>V56.8 Encounter for other dialysis<br>V56.1 Fitting and adj of extracorporeal dialysis<br>V56.2 Fitting and adj of peritoneal dialysis	N18.3 CKD stage 3<br>N18.4 CKD stage 4<br>N18.5 CKD stage 5<br>N18.6 ESRD<br>**Procedure codes**<br>I77.0 AV fistula<br>T82.590A AV graft placement<br>T85.621 Displacement of intraperitoneal dialysis catheter<br>T82.4 (including .41, .42, .43, .49) complication of vascular dialysis catheter<br>R880 Cloudy (hemodialysis) peritoneal dialysis effluent<br>Z99.2 Dependence on renal dialysis<br>Z94.0 Kidney transplant status<br>Z49.31 Encounter for hemodialysis<br>Z91.15 Noncompliance with renal dialysis<br>Z49.32 Encounter for peritoneal dialysis<br>Z49.01 Encounter for fitting and adj extracorporeal dialysis<br>Z49.02 Encounter for fitting and adj peritoneal dialysis	585.x	N18.%
Secondary outcomes				
**Any CKD**	585.1-585.6<br>*Plus, procedure codes listed above*	N18.1-N18.6<br>*Plus, procedure codes listed above*	585.x	N18.%
**CKD severe+ (stage 4-ESRD)**	585.4-585.6<br>*Plus, procedure codes listed above*	N18.4-N18.6<br>*Plus, procedure codes listed above*	585.x	N18.%

*For ICD-9-CM codes, "x" is used as a wildcard character, while for ICD-10-CM codes, "%" is used as a wildcard since "x" is a valid character in the ICD-10-CM system.

© 2025 Medgyesi DN et al. *JAMA Network Open*.

eTable 3. Trihalomethane (THM) data from community water supplies (CWS) serving the residential addresses of California Teachers Study participants. The data are grouped according to the monitoring timeframe. For years with missing data, we interpolated values by assigning the average of annual concentrations within the same period. If no data was available, we used the average from the closest period, either before (when available) or after, within the same monitoring timeframe. For pre-regulation years without data, we assigned the average from the first available period of Stage 1 monitoring. The table shows the percentage of CWS with annual concentrations within each period, as well as the concentration distribution before and after interpolation.

**Monitoring timeframe**		**N (%) CWS with annual measurement(s)[a]**		**Distribution total THMs (µg/L) before/after interpolation**				
	Period	**Before interpolation**	**After interpolation**	**Mean**	**Median**	**25th**	**75th**	**95th**
Pre-regulation	1990-1997	969 (68.4%)	1258 (88.8%)	4.9/4.5	0.2/0.2	0.1/0.1	3.5/1.7	27.6/28.3
**Stage 1**	1998-2001	1003 (70.8%)	1258 (88.8%)	5.6/5.3	0.2/0.2	0.1/0.1	2.0/1.2	34.4/34.1
	2002-2005	1164 (82.1%)	1258 (88.8%)	7.9/7.5	0.3/0.3	0.1/0.1	7.6/6.7	41.1/40.4
**Stage 2[b]** – initial	2006-2011	1287 (90.8%)	1287 (90.8%)	7.0/7.0	0.5/0.5	0.1/0.1	6.1/6.1	36.2/36.2
	2012	805 (56.8%)	1183 (83.5%)	16.6/13.5	2.9/1.3	0.2/0.1	30.3/21.4	56.8/53.9
	2013	891 (62.9%)	1183 (83.5%)	18.2/14.7	7.7/2.5	0.3/0.1	33.2/26.5	57.0/54.7
	2014	984 (69.4%)	1183 (83.5%)	18.3/15.8	8.8/4.8	0.9/0.2	31.6/28.0	60.0/56.3
	2015	970 (68.5%)	1221 (86.2%)	20.0/16.7	10.8/5.4	1.1/0.3	34.1/28.4	62.2/59.0
	2016	997 (70.4%)	1221 (86.2%)	19.7/16.7	11.2/6.8	1.4/0.3	33.8/29.3	58.8/56.7
	2017	1014 (71.6%)	1221 (86.2%)	18.1/15.7	11.6/7.5	1.3/0.3	30.4/27.0	54.0/51.6
	2018	1019 (71.9%)	1165 (82.2%)	18.2/16.9	11.6/8.9	1.2/0.6	31.5/28.6	54.0/53.6
	2019	1052 (74.2%)	1165 (82.2%)	18.8/17.4	13.2/10.5	2.2/1.0	31.2/29.4	53.5/52.6

[a]Percentages are of 1,417 total CWS serving residential addresses of participants throughout follow-up (1995-2018). 1,409 CWS had at least one trihalomethane measurement.
[b]The U.S. EPA's Stage 2 Disinfectants and Disinfection Byproducts Rule (DBPR) began in 2006. This regulation changed how MCL compliance was calculated—from a system-wide running annual average (RAA) to a locational running annual average (LRAA)—to better capture high-exposure sites within distribution systems. This shift likely contributed to the apparent rise in concentrations during that period.
Blue rows represent years of data used to calculate primary exposure period for this study (1995-2005). Years after were used to calculate cumulative moving averages for time varying analyses.

© 2025 Medgyesi DN et al. *JAMA Network Open*.

eTable 4. Correlation (Spearman's rho) between total, brominated and individual trihalomethanes (THMs), uranium and arsenic, and haloacetic acid concentrations. Average exposure from residential community water supplies (1995-2005) for participants in the California Teacher Study included in this analysis (n=89,320).

	**Trihalomethanes (THMs)**						**Metals[1]**		**Haloacetic acids (HAAs)[2]**					
	Total THMs	Brominated THMs	Chloroform	BDCM	DBCM	Bromoform	Uranium	Arsenic	HAA5	MCA	MBA	DCA	DBA	TCA
Total THMs	1													
Brominated THMs	0.86	1												
Chloroform	0.92	0.79	1											
Bromodichloromethane (BDCM)	0.90	0.97	0.88	1										
Dibromochloromethane (DBCM)	0.75	0.96	0.64	0.91	1									
Bromoform	0.59	0.80	0.42	0.68	0.83	1								
Uranium	-0.14	-0.01	-0.23	-0.10	0.06	0.16	1							
Arsenic	-0.31	-0.20	-0.35	-0.27	-0.14	-0.02	0.48	1						
Haloacetic acids, five (HAA5)	0.62	0.42	0.67	0.48	0.32	0.20	-0.28	-0.26	1					
Monochloroacetic acid (MCA)	0.19	0.06	0.27	0.09	-0.01	-0.03	-0.12	-0.29	0.37	1				
Monobromoacetic acid (MBA)	0.15	0.29	-0.01	0.26	0.35	0.26	0.17	0.00	0.06	0.19	1			
Dichloroacetic acid (DCA)	0.63	0.36	0.73	0.47	0.23	0.12	-0.06	-0.26	0.90	0.41	-0.05	1		
Dibromoacetic acid (DBA)	0.63	0.78	0.36	0.74	0.82	0.70	0.05	-0.25	0.50	0.16	0.30	0.40	1	
Trichloroacedic acid (TCA)	0.64	0.37	0.74	0.47	0.23	0.10	0.00	-0.06	0.91	0.28	0.01	0.89	0.30	1

[1]Avaliable for 88,169 participants
[2]Available for a subset of 32,277 participants

© 2025 Medgyesi DN et al. *JAMA Network Open*.

eTable 5. Further adjustments of the hazard ratios (HRs) and 95% confidence intervals (95%CIs) for chronic kidney disease (CKD) risk associated with average concentrations of total, brominated, and individual trihalomethanes (1995-2005) from residential community water supplies in the California Teachers Study (n=86,804; analytic follow-up=2005-2018). The number of years ≥ ½ the maximum contaminant level (MCL) shown for total trihalomethanes.

	n	cases	HR (95%CI)[a,b]
**Total trihalomethanes (µg/L)**			
Q1 (≤0.4)	20572	1316	1.00 (REF)
Q2 (0.5-5.4)	22911	1526	1.04 (0.97, 1.13)
Q3 (5.5-24.0)	21627	1523	1.10 (1.02, 1.19)
Q4 (24.1-57.7)	17087	1261	1.16 (1.07, 1.26)
≥95th (57.8-93.7)	4607	433	1.15 (1.02, 1.30)
ptrend[c]			0.001
Continuous IQR			1.07 (1.03, 1.11)
Years ≥ 1/2 MCL (40µg/L)[d]			
0 years	64208	4434	1.00 (REF)
1-3 yrs	9865	615	1.09 (1.00, 1.19)
4-11 yrs	12731	1010	1.11 (1.03, 1.20)
**Brominated trihalomethanes (µg/L)**			
Q1 (≤0.6)	20971	1284	1.00 (REF)
Q2 (0.7-2.6)	22267	1556	1.15 (1.07, 1.25)
Q3 (2.7-11.2)	21816	1545	1.08 (1.00, 1.17)
Q4 (11.3-29.9)	17296	1343	1.22 (1.12, 1.32)
≥95th (30.0-42.9)	4454	331	1.41 (1.21, 1.64)
ptrend[c]			<0.001
Continuous IQR			1.07 (1.04, 1.11)
**Individual trihalomethanes**			
**Chloroform (µg/L)**			
Q1 (≤0.2)	19817	1242	1.00 (REF)
Q2 (0.3-2.3)	23141	1552	1.05 (0.97, 1.14)
Q3 (2.4-9.0)	22166	1651	1.15 (1.06, 1.24)
Q4 (9.1-47.2)	17359	1205	1.11 (1.02, 1.21)
≥95th (47.3-84.1)	4321	409	1.12 (0.99, 1.27)
ptrend[c]			0.12
Continuous IQR			1.02 (1.00, 1.04)
**Bromodichloromethane (µg/L)**			
Q1 (≤0.2)	21103	1340	1.00 (REF)
Q2 (0.3-1.3)	22285	1528	1.06 (0.98, 1.15)
Q3 (1.4-5.7)	21606	1499	1.06 (0.98, 1.15)
Q4 (5.8-16.3)	17473	1371	1.14 (1.05, 1.23)
≥95th (16.4-21.1)	4337	321	1.32 (1.12, 1.54)
ptrend[c]			<0.001

© 2025 Medgyesi DN et al. *JAMA Network Open*.

Continuous IQR			1.07 (1.04, 1.11)
**Dibromochloromethane (μg/L)[e]**			
Q1-Q2 (≤0.7)	41894	2736	1.00 (REF)
Q3 (0.8-4.5)	23197	1670	1.03 (0.96, 1.10)
Q4 (4.6-12.0)	17341	1331	1.12 (1.04, 1.20)
≥95th (12.1-18.3)	4372	322	1.31 (1.13, 1.52)
ptrend[c]			<0.001
Continuous IQR			1.07 (1.04, 1.11)
**Bromoform (μg/L)[e]**			
Q1-Q2 (≤0.1)	38873	2661	1.00 (REF)
Q3 (0.2-1.4)	24892	1650	1.03 (0.96, 1.10)
Q4 (1.5-3.0)	18521	1421	1.08 (1.01, 1.16)
≥95th (3.1-18.1)	4518	327	1.21 (1.07, 1.36)
ptrend[c]			0.001
Continuous IQR			1.06 (1.02, 1.10)

[a]Adjusted for continuous BMI, smoking status, race and ethnicity, neighborhood SES quartiles, Census region as a random effect, and age as the underlying time scale.

[b]Additionally adjusted for self-reported, diabetes, hypertension, menopause status, exercise (hours/week), and smoking pack years (excluding 2,516 missing)

[c]Modeled as a continuous variable derived from the median of each exposure category

[d]Categorized by zero years of exposure, and further split by < or ≥ median years for those with at least one year of exposure at and above one-half the MCL threshold

[e]Reference of below the median was used because of the limited concentration distribution

© 2025 Medgyesi DN et al. *JAMA Network Open*.

eTable 6. Competing risk models including death as a competing risk of chronic kidney disease (CKD). Hazard ratios (HRs) and 95% confidence intervals (95%CIs) for CKD risk associated with average concentrations of total and brominated trihalomethanes (1995-2005) from residential community water supplies in the California Teachers Study (n=86,804; analytic follow-up=2005-2018).

	n	cases	deaths	HR (95%CI)[a]
**Total trihalomethanes (µg/L)**				
Q1 (≤0.4)	21144	1355	3159	1.00 (REF)
Q2 (0.5-5.4)	23550	1567	3424	1.04 (0.96, 1.12)
Q3 (5.5-24.0)	22256	1567	3260	1.07 (0.99, 1.16)
Q4 (24.1-57.7)	17592	1303	2556	1.14 (1.05, 1.24)
≥95th (57.8-93.7)	4778	450	813	1.12 (1.00, 1.27)
ptrend[b]				0.003
Continuous IQR				1.06 (1.02, 1.10)
**Brominated trihalomethanes (µg/L)**				
Q1 (≤0.6)	21569	1319	3223	1.00 (REF)
Q2 (0.7-2.6)	22892	1603	3295	1.14 (1.06, 1.23)
Q3 (2.7-11.2)	22477	1593	3452	1.04 (0.96, 1.13)
Q4 (11.3-29.9)	17783	1387	2660	1.20 (1.11, 1.31)
≥95th (30.0-42.9)	4599	340	582	1.37 (1.18, 1.60)
ptrend[b]				<0.001
Continuous IQR				1.06 (1.03, 1.10)

[a]Adjusted for age, continuous BMI, smoking status, race and ethnicity, neighborhood SES quartiles, and Census region.
[c]Modeled as a continuous variable derived from the median of each exposure category

© 2025 Medgyesi DN et al. *JAMA Network Open*.

eTable 7. Time-varying analyses of cumulative moving average total and brominated trihalomethane exposure from residential community water supplies since 1995. Cumulative moving average also lagged by 5-years. Hazard ratios (HRs) and 95% confidence intervals (95%CIs) for moderate+ chronic kidney disease (CKD) risk in the California Teachers Study (n=89,320; analytic follow-up=2005-2018).

	Cumulative moving average[a]				Cumulative moving average, lagged 5 years		
	person years	cases	HR (95%CI)[b]		person years	cases	HR (95%CI)[b]
**Total trihalomethanes (µg/L)**							
Q1 (≤1.7)	266017	1107	1.00 (REF)	Q1 (≤0.4)	255199	1105	1.00 (REF)
Q2 (1.8-9.8)	268147	1684	1.32 (1.23, 1.43)	Q2 (0.5-6.3)	278555	1650	1.24 (1.15, 1.33)
Q3 (9.9-23.9)	267901	1723	1.31 (1.21, 1.42)	Q3 (6.4-23.5)	266824	1775	1.33 (1.23, 1.44)
Q4 (24.0-57.6)	214361	1292	1.33 (1.22, 1.46)	Q4 (23.6-56.7)	212757	1260	1.26 (1.16, 1.37)
≥95th (57.7-102.2)	53392	436	1.37 (1.21, 1.56)	≥95th (56.7-127.9)	56483	452	1.39 (1.23, 1.57)
*p*trend[c]			<0.001	*p*trend[c]			<0.001
Continuous IQR			1.09 (1.05, 1.13)	Continuous IQR			1.07 (1.04, 1.11)
**Brominated trihalomethanes (µg/L)**							
Q1 (≤0.8)	251747	1151	1.00 (REF)	Q1 (≤0.4)	249857	1022	1.00 (REF)
Q2 (0.9-4.5)	280316	1570	1.17 (1.08, 1.27)	Q2 (0.5-2.5)	279099	1718	1.36 (1.26, 1.47)
Q3 (4.6-12.0)	269422	1830	1.28 (1.17, 1.39)	Q3 (2.6-11.0)	271802	1730	1.32 (1.22, 1.44)
Q4 (12.0-29.1)	214472	1375	1.31 (1.20, 1.43)	Q4 (11.1-30.0)	215213	1484	1.45 (1.33, 1.58)
≥95th (29.2-66.4)	53861	316	1.48 (1.27, 1.73)	≥95th (30.1-61.8)	53847	288	1.41 (1.21, 1.65)
*p*trend[c]			<0.001	*p*trend[c]			<0.001
Continuous IQR			1.10 (1.06, 1.14)	Continuous IQR			1.08 (1.04, 1.11)

[a]Cumulative moving average since the start of the exposure period (1995), recalculated for each year of follow-up until the time of a CKD event, move out of California, death or the end of follow-up (12/31/2018)

[b]Adjusted for continuous BMI, smoking status, race and ethnicity, neighborhood SES quartiles, Census region as a stratification term, and age as the time scale. Participant ID also included as a cluster term to account for multiple observations per participant in time-varying analyses.

[c]Modeled as a continuous variable derived from the median of each exposure category

© 2025 Medgyesi DN et al. *JAMA Network Open*.

eTable 8. Stratified analyses by baseline age, body mass index (BMI), and smoking status in the California Teachers Study (n=89,320; analytic follow-up=2005-2018). Hazard ratios 95% confidence intervals (95%CI) for moderate+ chronic kidney disease (CKD) risk associated with per IQR average total and brominated trihalomethane exposure (µg/L) from residential community water supplies (1995-2005).

	n (cases)	Total THM, IQR HR (95%CI)	p-int.	Brominated THM, IQR HR (95%CI)	p-int.
Overall	89320 (6242)	1.07 (1.03, 1.11)		1.08 (1.04, 1.11)	
Age (years)			0.34		0.28
≤55	57311 (1669)	1.05 (0.98, 1.13)		1.05 (0.99, 1.12)	
>55	32009 (4573)	1.07 (1.03, 1.12)		1.09 (1.05, 1.13)	
BMI (kg/m²)			0.98		0.82
<25	54450 (2592)	1.07 (1.01, 1.13)		1.09 (1.03, 1.14)	
≥25	34870 (3650)	1.07 (1.02, 1.12)		1.07 (1.02, 1.11)	
Smoking status			0.68		0.16
Never	59546 (3679)	1.07 (1.02, 1.12)		1.06 (1.02, 1.10)	
Ever	29774 (2563)	1.07 (1.01, 1.13)		1.10 (1.04, 1.15)	

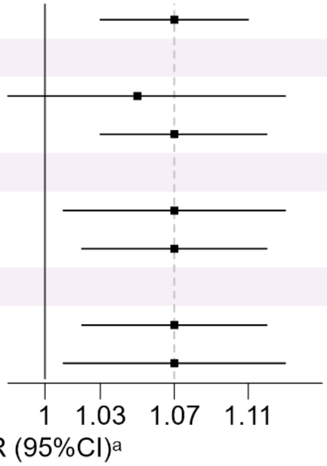

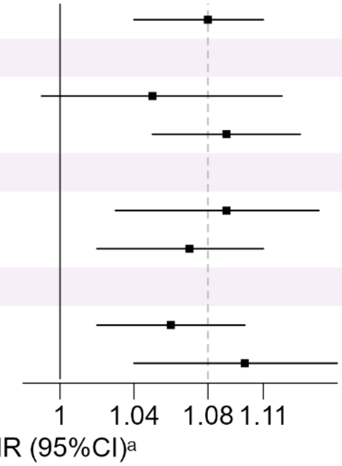

[b]Adjusted for continuous BMI, smoking status (except for stratified model by smoking status), race and ethnicity, neighborhood SES quartiles, Census region as a random effect, and age as the underlying time scale.
p-int.: *p*-value for interaction computed as a cross-product between continuous exposure and binary stratification term
Dashed line denotes the overall hazard ratio

© 2025 Medgyesi DN et al. *JAMA Network Open*.

eTable 9. Hazard ratios (HRs) and 95% confidence intervals (95%CIs) for chronic kidney disease (CKD) risk associated with average concentrations of haloacetic acids (1995-2005) from residential community water supplies in the California Teachers Study (n=33,277; analytic follow-up=2005-2018). Exposures categorized into tertiles.

	n	cases	HR (95%CI)[a]
**Haloacetic acids, five (HAA5) (µg/L)**			
T1 (≤1.39)	10475	907	1.00 (REF)
T2 (1.40-21.9)	11138	771	0.97 (0.84, 1.11)
T3 (22.0-90.9)	10664	795	0.99 (0.85, 1.16)
*p*trend[b]			0.79
Continuous IQR			0.99 (0.89, 1.10)
**Dichloroacetic acid (µg/L)**			
T1 (≤2.49)	10317	710	1.00 (REF)
T2 (2.50-9.79)	10305	695	0.95 (0.85, 1.07)
T3 (9.80-43.3)	10740	793	0.99 (0.88, 1.12)
*p*trend[b]			0.95
Continuous IQR			0.98 (0.90, 1.06)
**Trichloroacetic acid (µg/L)**			
T1 (≤1.49)	10216	700	1.00 (REF)
T2 (1.50-6.89)	8671	608	0.97 (0.86, 1.10)
T3 (6.90-110.0)	12478	890	0.94 (0.83, 1.07)
*p*trend[b]			0.36
Continuous IQR			1.01 (0.95, 1.07)
**Dibromoacetic acid (µg/L)**			
T1 (≤0.79)	10311	651	1.00 (REF)
T2 (0.80-4.19)	10675	774	1.12 (0.97, 1.28)
T3 (4.20-67.0)	10376	773	1.09 (0.93, 1.28)
*p*trend[b]			0.75
Continuous IQR			1.05 (0.97, 1.14)
**Monobromoacetic acid (µg/L)**			
T1 (≤0.19)	2830	171	1.00 (REF)
T2 (0.20-0.29)	8201	538	0.94 (0.78, 1.14)
T3 (0.30-13.0)	20331	1489	0.98 (0.83, 1.15)
*p*trend[b]			0.99
Continuous IQR			1.00 (0.98, 1.02)
**Monochloroacetic acid (µg/L)**			
T1 (≤0.09)	5420	355	1.00 (REF)
T2 (0.10-0.39)	14640	1009	0.87 (0.76, 0.99)
T3 (0.40-8.30)	11302	834	0.90 (0.78, 1.04)
*p*trend[b]			0.96
Continuous IQR			0.98 (0.95, 1.01)

[a]Adjusted for continuous BMI, smoking status, race and ethnicity, neighborhood SES quartiles, Census region as a random effect, and age as the time scale.
[b]Modeled as a continuous variable derived from the median of each exposure category

© 2025 Medgyesi DN et al. *JAMA Network Open*.

133,477

Residential and administrative exclusions[1]
Baseline address outside California (n=8,795)
PO boxes and poor geocode quality (n=5,989)
Breast cancer research only (n=16)

118,692

Study-specific and exposure exclusions
Lived outside CWS; private well users (n=9,441)[2]
Moved out of California or died before 2005 (n=14,901)
Missing contaminant measurements (n=611)

93,739

CKD exclusions
Prevalent CKD (n=73)
Age ≥85 years at enrollment (n=567)

93,099

Main covariate exclusions
n=3,779

89,320

**Moderate+ CKD**	**6,242 (7.0%)**
All stages CKD	8,846 (9.9%)
Advanced+ CKD	1,505 (1.7%)

[1]Medgyesi DN, Spielfogel ES, Ward MH, Jones RR, Savage KE, Benbow JL, Lacey JV, Sanchez TR. Construction of residential histories to estimate long-term environmental exposures in the California Teachers Study cohort. J Expo Sci Environ Epidemiol. 2023. 10.1038/s41370-023-00631-0
[2]This exclusion applied to anyone who lived outside of community water supply (CWS) boundaries at any point during the exposure period (1995-2005). 6,713 (71% of excluded participants) lived outside boundaries at enrollment.

eFigure 1. Inclusion flowchart for analysis of trihalomethane exposure from community water supplies and chronic kidney disease risk in the California Teachers Study.

© 2025 Medgyesi DN et al. *JAMA Network Open*.

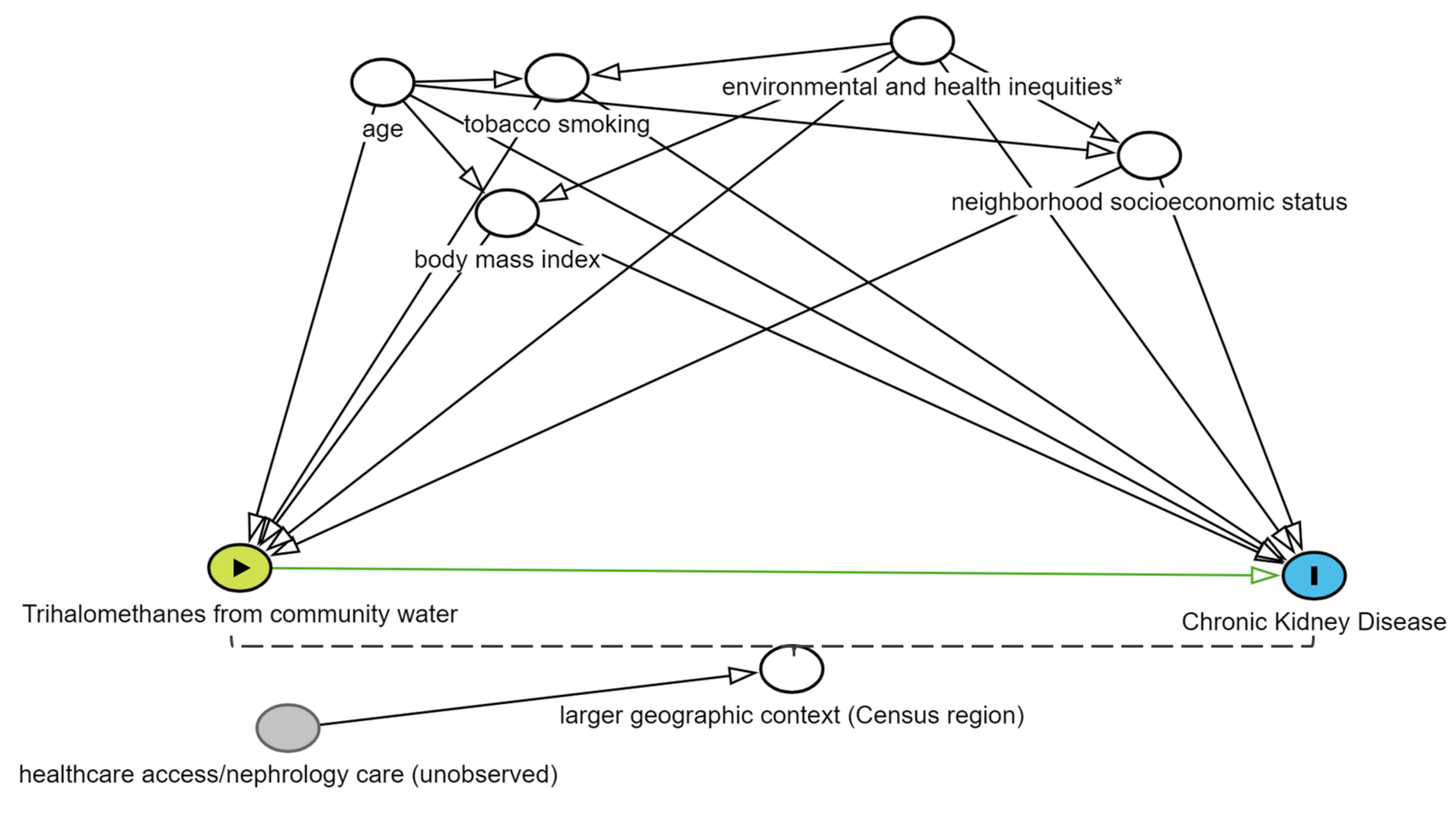

eFigure 2. Directed acyclic graph (DAG) conceptualizing potential confounding factors between trihalomethane exposure from community water supplies and the development of chronic kidney disease (CKD. The influence of region is captured as a random effect. Age is used as the underlying time metric. *We adjusted for race and ethnicity because environmental and health inequities that affect racial and ethnic groups, arising from social factors including historic redlining and disparities in healthcare access, can contribute to greater exposure to drinking water contaminants and an increased risk of CKD. Drawn using https://www.dagitty.net/

© 2025 Medgyesi DN et al. *JAMA Network Open*.

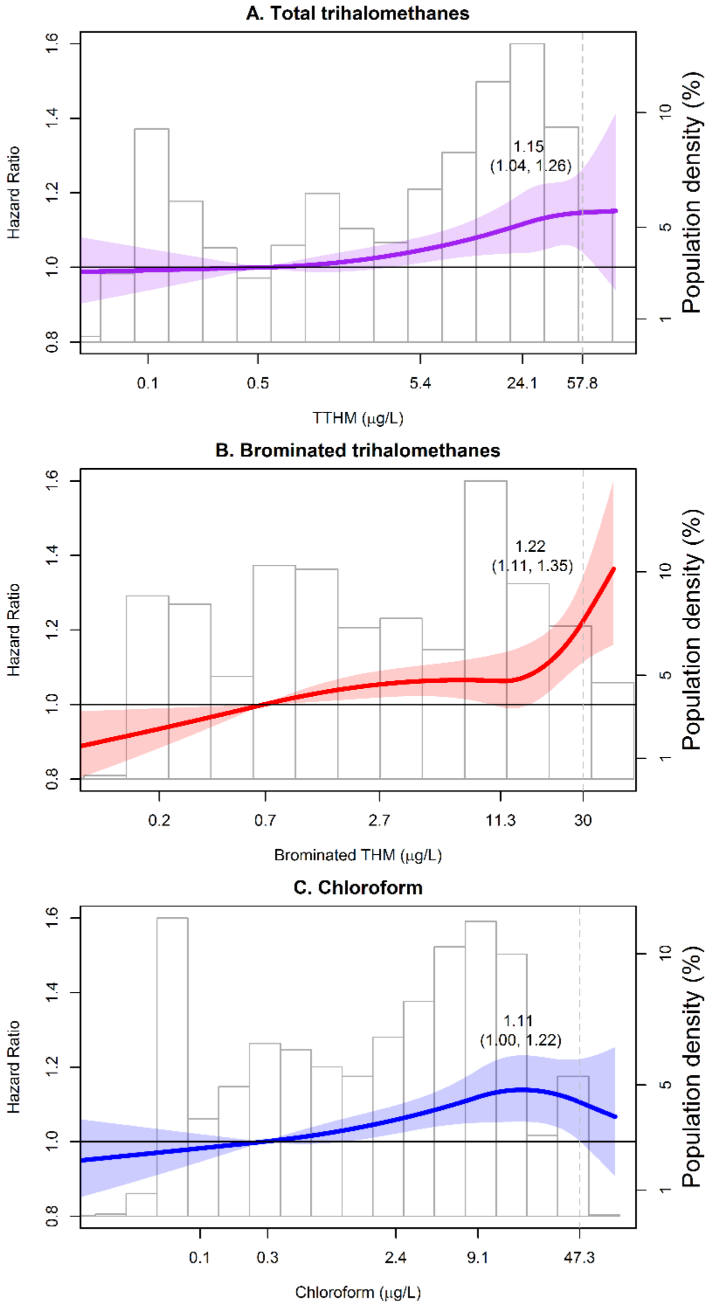

eFigure 3. X-axis on the log-scale with labels at the 10th, 25th, 50th, 75th, and 95th percentiles. Hazard ratios (HRs) and 95% confidence intervals (95%CIs) for moderate+ chronic kidney disease (CKD) risk associated with average a) total trihalomethanes, b) brominated trihalomethanes, and c) chloroform (1995-2005) from residential community water supplies modeled as restricted cubic splines in the California Teachers Study (n=89,320; analytic follow-up=2005-2018). HRs were adjusted for continuous BMI, smoking status, race and ethnicity, neighborhood SES quartiles, Census region as a random effect, and age as the time scale. Concentrations were log-transformed with knots placed at the 25th, 75th and 95th percentiles. Specific HRs (95%CIs) are provided at the 95th percentile (total trihalomethanes: 57.8μg/L, brominated trihalomethanes: 30.0μg/L, chloroform=47.3μg/L) in reference to the 25th percentile (0.5μg/L, 0.7μg/L, 0.3μg/L, respectively). A histogram of the population density is shown behind each spline.

© 2025 Medgyesi DN et al. *JAMA Network Open*.
