# Supplementary material for: Community Water Trihalomethanes and Chronic Kidney Disease
Source: JAMA Netw Open. 2025 Jul 2;8(7):e2518513. doi: 10.1001/jamanetworkopen.2025.18513 (PMC12223891; doi:10.1001/jamanetworkopen.2025.18513)
Supplement: Supplement 2. — Data Sharing Statement [file jamanetwopen-e2518513-s002.pdf]

Data Sharing Statement

Medgyesi. Community Water Trihalomethanes and Chronic Kidney Disease. *JAMA Netw Open*. Published July 02, 2025. doi:10.1001/jamanetworkopen.2025.18513

Data
Data available: No

Additional Information
Explanation for why data not available: All of the data associated with this publication and in the California Teachers Study are available for research use. The California Teachers Study welcomes all such inquiries and encourages individuals to visit https://www.calteachersstudy.org/for-researchers.
